# Dr. Seymour on the Sedative Treatment of Insanity

**Published:** 1848-01-01

**Authors:** 


THE JOURNAL

OF

PSYCHOLOGICAL MEDICINE

AND

MENTAL PATHOLOGY.

JANUARY 1, 1848.

### Analytical Reviews.

ART. I.—*On Mental Derangement.* By EDWARD J. SEYMOUR, M.D., F.R.S. &c. &c. (Forming a chapter in his Work entitled, "Thoughts on the Nature and Treatment of several severe Diseases of the Human Body." In 2 vols. 8vo. Vol. i. 1847. Longman and Co.)

THE late Sir Henry Halford was something more than an accomplished courtier. Those who had the pleasure of this distinguished physician's personal acquaintance, and who had opportunities of hearing from his own lips his opinions of "men and things," could not have been otherwise than struck with his sagacity on all matters to the consideration of which he had directed his able mind. It is well known that the subject of insanity was one of Sir H. Halford's favourite topics of study. We well recollect a long and deeply interesting conversation which we had with him, on the various points connected with the question of disordered mind, during which he showed evidence of having paid great attention to the treatment of the insane. He entertained a strong opinion—which opinion he expressed most emphatically, when examined before a committee of the House of Commons—of the importance of paying greater attention than medical men, having specially to do with the insane, were disposed, to the medical treatment of insanity. Without wishing to undervalue the great importance of an efficient system of moral treatment, he considered that *great things* were only to be expected from a persevering administration of those powerful remedial agents whose virtues are made the subject of such high encomiums in the pages of the Materia Medica.

Sir H. Halford was right in his opinion. We are quite aware that many distinguished men, experienced in the management of the insane, have little faith in purely medical treatment. We think they under-estimate the efficacy—often great efficacy—of medicine in the cure of those affections of the brain which derange the manifestation of the mind. We have witnessed, in the course of our practice, the most marked advantage, in cases apparently hopeless, from a persevering use of physical means in the cure of insanity; in fact, we never give up a case until symptoms indicate that the patient is beyond the reach of recovery. Even in the worst and most incurable cases the symptoms may often be much mitigated, and the condition of the patient greatly improved, by a patient application of the means placed within our power.

Foremost among the distinguished men of the present day who have paid particular attention to the medical treatment of mental derangement stands the name of Dr. Seymour. We recollect reading, with much pleasure and profit, a treatise published many years ago by him on this subject. This work is now out of print. In the essay before us, he has given us the benefit of his additional experience of the influence of medicine in the cure of insanity. We pronounce, without hesitation, this essay to be one of the most important contributions to the therapeutics of insanity which has appeared in this country for many years. We should have predicated, from the frequent opportunities which Dr. Seymour has had of making himself thoroughly conversant with the treatment of the insane, from the character of his mind, and profound knowledge of every department of medicine, that he, of all men in the profession, was most competent to give us an insight into the influence of medicinal agents in the cure of disorders of the mind. His essay has realized our best hopes. It is full of most valuable suggestions, and is written in a most pleasing yet philosophic style. The author has, in the course of his long and useful career, filled many high, honourable, and responsible posts. Independently of his long connexion as senior physician to St. George's Hospital, he officiated for the period of eight years as one of the Metropolitan Commissioners in Lunacy, and during that time had constant opportunities of visiting establishments for the reception of the insane, and of becoming most intimately acquainted with the many phases of this most strange and mysterious malady. "To *see* is not to *observe*," says a quaint authority. It is fortunate for the cause of humanity that Dr. Seymour is endowed with great powers of observation, and that he has not only seen, but observed; and we have now before us the result of the reflections of his highly-gifted mind on a subject of all others the most difficult of comprehension.

The essay on Mental Derangement forms a chapter in a work entitled, "Thoughts on the Nature and Treatment of several severe Diseases of the Human Body." The whole work is full of valuable information.

Dr. Seymour's essay refers particularly to that form termed melancholia, or the alternation of melancholia with violence, termed lypomania, or suicidal madness, especially in reference to the treatment of this form of disease by the employment of opiates, or of that class of medicines called sedatives.

The author refers to two morbid actions of the brain—the one, in which the functions of the encephalon are increased in power, producing a violent succession of ill-regulated mental actions; and the other, in which perception and volition are greatly reduced in force, giving rise to debility of mind sometimes amounting to idiocy.

Dr. Seymour conceives that this inordinate action and impairment, or reduced power of mind, may take place as a functional disorder only, independently of any actual disease of the structure of the brain itself.

On this point we do not entirely agree with Dr. Seymour. We are more disposed to side with those, who maintain that the disorders of the mind are but the results of some derangement in the *physical* structure of the brain and nervous system, and that such a thing as a purely functional disorder of the intellect is a pathological impossibility.

Independently of other considerations, we consider this view of the matter as the most consolatory to the physician called upon to administer, as the phrase is, " to the mind diseased." If the nervous fibre of the brain, the recognised organ of mind, remains intact during the ravages of mental disorder, upon what do we purpose acting with our medicinal agents ? Can we imagine such a thing as a purely functional disease of any organ, setting aside the brain altogether ? We opine not. We do not maintain, that in every affection of the mind coming within the category of mental derangement, the nervous structure is broken down, and that, after death, palpable and obvious marks of organic disease can invariably be detected. We know that, in many instances of death from long continued insanity, even in its worst forms, no lesions of nervous matter are perceptible by the naked eye in the post mortem examination. Even in these cases, we do not think that we are altogether justified in concluding the absence of any appreciable alteration in the sensorium as conclusively establishing the existence of a disorder purely functional in its character. We know little or nothing of the intimate nature of nervous matter, and still less of the influence of certain diseased modifications of the blood and nervous principle on the brain and manifestations of the mind, and until these points are satisfactorily investigated, we must exercise caution in giving an opinion as to the precise relationship between diseased structure and disordered function.

It is not our purpose on this occasion to discuss, in all its complexity, this *vexata quæstio;* it is sufficient to say, that on this point we differ from the learned author of this essay.

The following passage conveys Dr. Seymour's views as to the pathological state of the brain in insanity :—

" Where perception and volition are sharpened beyond all measure, there are remedies which quiet pain and diminish sensibility, in many cases not absolutely removing the pain, but preventing the brain from perceiving it in a highly aggravated form : thus, in paroxysms of gall-stones, or sciatica, or in nephritic colic, the pain is removed partly by the solution of spasm afforded by the remedies, partly from the increased sensibility or perception of the brain being diminished.

" In inordinate actions, also, the causes being investigated, appropriate remedies may be found : such as the shower bath, metallic tonics, and the like.

" Mental derangement is one of these forms of increased, or irregular, or diminished perception and volition, and it exists in some instances secondarily to diseases of organs at a distance, as of the uterus or stomach, or in cases of fever from the poison of the atmosphere, or putrid miasmata from different sources. The brain, like other organs of the body, is liable to inflammation, and which requires to be treated as inflammation is treated in other parts of the body ; and it is singularly liable, from the particular arrangement of its blood-vessels, to disturbance of the circulation ; either the blood not finding its usual ready flow into the right side of the heart, from some obstacle in the complicated arrangement of its veins (sinuses), or from disease of its arteries, which unite

(anastomose) one with another, in a most remarkable manner. The diseases of the coats of these vessels give rise to some of the most serious diseases of the brain

"As life advances, that portion of the human body most influenced by physical laws tends first to decay. The elasticity of the larger arteries becomes diminished, by, first, soft fatty matter (termed atheroma) deposited under the inner coat, or by bone itself. The tube, previously elastic, and easily and equally distended by the current of blood, becomes unequally distended, and even brittle in parts, so as to give way under any considerable or sudden acceleration of the circulation. When such an accident occurs in the brain, blood is thrown out, the patient becomes senseless, and when proper remedies have been applied, awakens as from a trance, with much confusion and distress, and often pain in the head, and finds the arm and leg, on the opposite side to that in which the vessel had burst, deprived of motion, and often, in a great degree, of sensation.

"An apoplectic seizure has terminated in palsy!

"Hence it is not necessary for this to occur simply from an over supply of blood in the system, and explains why it frequently happens in poor and spare habits of body, provided that the change noticed in the arterial system has commenced.

"I only allude to this, however, to show that the part of the brain where these accidents occur may be seriously injured, without (beyond the first moments of the attack) deranging the mind. If the blood be poured out in small quantity, the attack is recovered from in a greater or less proportional space of time; but the palsy remains for years, showing the severe injury sustained. Notwithstanding this, mind, perception, and volition, are not injured, (at least in by far the greater number of instances); the patient soon reads and understands as usual, gives his orders, takes his exercise, performs his religious duties with quite equal intelligence to that which he possessed previous to his illness. By some this has been explained, that the brain is a double organ; but the experiments of Fontana and others have proved that a very large portion of both hemispheres of the brain may be removed without any visible disturbance of the intellect of the animal before inflammation ensues.

"If, however, a similar accident, rupture of an artery, occurs in the cerebellum, or at the base of the brain, life becomes almost immediately extinct.

"The process of destruction which has occasioned the disease which we commented on, may also go on in the minute branches of arteries in the brain, and then a softening or pulpy condition of the brain ensues, gradually extending. If this be in the posterior portion of the brain, various accidents occur; palsy of the lower extremities, giddiness, deafness, insecurity in walking, and by degrees the mental powers become diminished; perception in a great degree fails, volition is imperfect, the memory is much impaired; and these symptoms gradually increase until death closes the scene.

"Thus, lesions of the important portions of the brain where the nerves of sense arise, or injuries of the cerebellum, are almost immediately recognisable by their severe effects on the body.

"Such a condition, if united with mental derangement, is necessarily fatal. Here we have seen one portion of the brain greatly injured without the mind suffering, and in another the mind gradually becoming obscured, especially as to memory, by destruction operating in another part of the brain. Tumours, exostoses of the skull, malignant disease, (fungus hæmatodes,) tubercles, all exist likewise in the brain of lunatics, but they also exist frequently in the brain, and go on to produce disease in distant parts of the body, without mental derangement being the consequence.

"If, then, there is no evidence of morbid growth or change existing, marked by palsy (especially of the lower extremities), fits, loss of memory, impaired vision, deafness, &c., we may fairly believe that the mental derangement is the result of disturbance of the functions of the brain, either originally, or secondary to disease of some important organ at a distance, and we are bound by every sense of duty, by every reason which ought to direct the conduct of the physician, to apply the resources of our art to its cure."—Pp. 149—153.

In 1831, Dr. Seymour delivered, before the Royal College of Physicians, his Croonian lectures, in which he dwelt upon the advantages derived from the use of the *acetate of morphia* in the treatment of bad, and especially suicidal cases of mania. His views were published at length, fifteen years back, in the work already alluded to.* From that time to a recent

* "The Medical Treatment of Insanity."

period, Dr. Seymour has been in the habit of administering the same medicine, anxiously watching the result of cases. He considers no patient as "recovered," unless two years of unabated health have elapsed since the treatment concluded. Dr. Seymour has tested the efficacy of this medicine in many cases, and it must afford a high amount of gratification to his mind to be enabled to say, as he does in the volume before us, that during the last fifteen years "*upwards of seventy cases have recovered!*"

The sedative treatment of insanity was not unknown to the ancients. The Romans raised a statue to Musa, the physician of the Emperor Augustus, for curing his sovereign of melancholy by the use of the extract of lettuce.

With reference to the disuse of opium in the treatment of insanity, Dr. Seymour observes, with great truth—

"Why such medicines became entirely rejected in England, may be explained by the number of asylums kept by persons without any knowledge of the medical art, and the universally inculcated opinion, that moral means, and moral means alone, are all-sufficient, and the use of medicine restricted to the ordinary maladies or derangements of the human body, independent of the disorder, or disease, as the case may be, of the brain.

"Partly, also, because when lunatics are collected together on a large scale, namely, pauper lunatics, many of whom are farmed at from six to nine shillings a week, according to the poverty of the parishes, it would deprive the owners of these establishments of all possible profit, if medicine were to be *systematically employed*, *in order to cure after many weeks and months of treatment.*

"How could this be done on from six to nine shillings a week, including meat and clothing? Partly also from the theory, which traces all excitement to inflammation, in the cases of mania, and to the no less erroneous, but still more frequent error, that melancholia depended solely on disease of the structure, or at least of disorder of the functions of the liver, as a primary cause!"—P. 158.

We entirely concur in the view which Dr. Seymour has taken. Many instances of insanity have come under our own observation, in which the various preparations of opium have been recommended and tried without producing any marked effect upon the malady. Upon investigating the cases, we have almost invariably found that the medicine was never perseveringly administered; in fact, *the system was not brought under its sedative influence.* We do not assert, that in all cases of insanity the *acetate of morphia* will invariably cure, or even mitigate the symptoms of the malady. Cases occur in which the preparations of opium would have a most injurious effect upon the patient. Dr. Seymour does not, however, recommend its indiscriminate administration: he points out very clearly the description of case in which its exhibition is admissible. He is in the habit of recommending a solution of the acetate of morphia, forty drops of which contain one grain of the alkaloid salt.

With reference to the mode of administering this preparation, the Doctor says—

"The preparation which I have preferred, and, with two or three exceptions, I have always used, is the acetate of morphia; the mode of preparation the solution; forty drops of the solution which I have generally employed contain one grain of the alkaloid salt.

"It has generally been, in mild cases, my practice to begin by a quarter of a grain every night, in solution; then, after a week, to increase this to half a grain: it has rarely in such cases been necessary to increase the dose beyond half a grain. In severe cases I begin with half a grain (twenty drops), and increase it speedily to a grain (forty drops); rarely, most rarely, beyond this dose. The medicine is given at bed-time, and only at bed-time, the period which is intended for sleep; but it must be repeated, without the *intermission* of a single night, for several weeks in mild cases, for at least three months in the most severe ones.

"In some of these cases at first sleep is not produced; in very few *rest* is not produced. Slight nausea, and disturbance of the head, are felt the first few mornings, but in these cases almost always at first, and *always after* a short time, both sleep is procured and the waking hours are free from pain."—Pp. 159—160.

Dr. Seymour endeavours to explain the *modus operandi* of morphia on the brain and nervous system. He says—

"Suppose a man toiling with professional anxieties, and with domestic cares, returns home, after a larger proportion than usual of the annoyances of his profession or calling, fatigued beyond his powers, wearied in mind. He returns to rest unhappy, discontented, inveighing against his lot, and what he considers to be his peculiar cares. He sleeps sound, and when about to rise in the morning, the sun streaming in at the windows, after a sound sleep, how does he look upon the evils of the preceding day? do they not lose a large portion of their affliction? does he not look in a totally different point of view at the very causes of distress which afflicted him the night before?

"And this is precisely what the effect of morphia, properly employed, effects in cases of melancholy mental derangement, but not once or twice, as would be the case in trifling distress. Hence it must be repeated regularly every night until the nervous system is soothed!

"Thus it requires weeks for the medicine to be repeated regularly, even without a single night's intermission, and the cure, as I shall show by examples, is the result."—P. 160.

Many objections have been raised against a long-continued use of opium in mental affections, on the ground that the habit of opium eating would be formed, of which it would be difficult for the patient, when restored to health, to break himself. Upon this point, the author justly observes—

"If a man suffer from continued fever, or from diseases in which large evacuations have been necessary early in the disease, afterwards, when convalescent, what is the recommendation of the physician? Moderate and nutritious diet, quina, and one, two, or three glasses of wine in the twenty-four hours. Such a course repeated for several weeks entirely restores the patient's health, his muscular powers, &c.; but what should we say, if he increased the wine every day by a glass, in order to enjoy the pleasurable sensations? We should say he would become a drunkard, and deplore such an event. Yet this is precisely the argument used against the employment of opiates in this disease. Is it not reasonable (I am sure it is true), that in broken states of the nervous system, the regular use of these medicines in given doses, should succeed in restoring the nervous power, as wine and stimulants, and diet, did, in the former case, the muscular system?"—P. 161.

Dr. Seymour has not, in the course of his practice, seen a single case, either where the treatment has succeeded or where it has failed, in which the smallest injury has been done by the use of morphia, or where the habit of taking sedatives has been established after a cure has been effected.

In confirmation of his views, Dr. Seymour cites the particulars of several very interesting cases. We quote two of great interest:—

"CASE I.—A lady, aged about forty-three, was attacked in the month of August, 1833, (after exposure to severe distress by the death of a relation, who expired in her presence) with mental derangement.

"Her usual habits of thinking were those of great deliberation; she had lived much in society, and from her station mixed much in the world; nor had there at any time been any even the slightest indication of eccentricity or weakness of mind. Her mind was at the time I saw her filled with gloomy ideas: imaginary neglect of great and solemn duties, and a belief of having committed indescribable and even ill-defined crimes, constituted the principal features of her malady.

"Her bodily health was unusually robust, and she had scarcely ever suffered even from trifling bodily ailment.

"In the first instance the patient was bled, and took repeated doses of purgative medicines, but without any beneficial effect. The pulse was not weak; the nights were sleepless, and there was no pain in the head, but a sense of weight was described, and there was restlessness of the body always present, so that the patient would often endeavour to jump out of bed and run out of the room. During an unavoidable absence from London the patient was seen by my friend Dr. Southey, and she was kept under nauseating doses of tartar emetic without any satisfactory result. It was now resolved to try the morphia, and a grain of the acetate was ordered to be taken every night, the bowels to be kept open by small doses of castor oil.

"The severity of the symptoms became greatly diminished, and it occurred to me that the sedative effect of cold would greatly assist the operation of the medicine. Ice was therefore kept to the head in a bladder day and night. The morphia never failed to procure a good night; and thus by degrees, without any other remedy except those mentioned, the mind cleared up.

"The use of ice was gradually abandoned, but the morphia continued to be administered every night during three months, although all trace of insanity had disappeared six weeks from the commencement of the remedies, during ten days of which the ice was kept continually to the head. No relapse whatever has occurred in the patient.

"Case II.—A young lady had visited for her health and her husband's the baths of Kissengen, without relief: on their return the complaints of the husband became worse, and he died.

"The wife, wearied with nursing, tired from her journey, showed, previously to her husband's death, signs of mental derangement. She was removed to another hotel by her mother, and attended by a late physician and general practitioner of eminence. She was very melancholy, occasionally interrupting her delusions with hymns, or scraps of sacred poetry; occasionally, as is often the case, loquacious, and then again subsiding into absolute silence and idiotcy as regarded those around her. At the expiration of ten days, her medical attendants reported to their patient's mother, that it was a rule in such cases, that when the patient did not immediately recover, she ought to be removed to an asylum or a private lodging, attended only by nurses, and without the visits of the family. The lady to whom this was addressed was very indignant at this opinion being pressed, and dismissed those who offered it. In the evening of the same day I was sent for, without any information of what had passed. I saw the patient, and recommended twenty drops (half a grain) of morphia at bed-time, first opening the bowels. This was repeated every night for a fortnight; in eight days the patient was well enough to be informed of the death of her husband; at the end of a fortnight she accompanied her mother to her seat in the country. Three years have nearly elapsed, and no return or disposition to return has manifested itself.

"A few days after being called to the case, I was assisted by a medical man of great respectability, who came to London on purpose, when he heard strong measures had been recommended for one whom he had attended since her infancy."—Pp. 164—166.

In ordinary melancholy, in that form of melancholia termed hypochondriasis, as well, and in the insanity which arises immediately after parturition, the acetate of morphia has a marked beneficial effect. The following are the particulars of a puerperal case which yielded to this potent drug:—

"About nine years ago I was sent for to Islington to see a lady, who had been confined the day before. This lady had had, a short time before her expected time, a fright from an alarm of fire, and her labour, although some days after the fright, was believed to be on the whole premature: the child was living. The patient's state was dreadful; her screams and cries were under the apprehension that she was condemned, and in hell fire. The lochia had ceased to flow; the patient, young and always delicate, seemed to have almost superhuman force; but her expression and violence of fear were scarcely to be endured. The pulse was 130, or even quicker. I recommended a grain of the acetate of morphia in solution, and a warm bath, every evening; her bowels to be kept open. I did not visit her again for two or three days, and I well remember the astonishment with which the anxious friends assured me she was so much better.

"The practice was continued, and I only saw her once afterwards; the further attendance of a physician being quite unnecessary."—P. 173.

In melancholy insanity obviously associated with uterine derangement, in which the mental affection first manifests itself in the early months of pregnancy, and is generally the result of a fright or violent emotion of the mind, the preparations of opium have a marked beneficial influence. In this description of derangement, the patient accuses herself of having committed some crime; dreads the approach of a calamity to herself, husband, or children; refuses food, from a suspicion of poison being mixed with it; often manifests a suicidal tendency, and frequently exhibits an unnatural horror of those that she used most tenderly to love. Illustrative of this kind of case, the following particulars are given:—

"I was requested in the month of September, 1838, to visit a lady professionally. The history of her case was as follows:—Her age was about thirty-two; she was the mother of several children; when in her pregnancy, advanced about three months, she was visiting in the house of a near relation, where a death took place after a very short illness. Peculiarly susceptible amidst the distress of all around her, it made a profound impression, not perhaps at the moment; its effects were not visible for about a month, when symptoms of mental aberration showed themselves. The care of her friends wasted time as well as they could, in hopes the accouchement would act as the resolution of the malady. The labour was short, and as is usual in such cases the pains made little impression on the patient; but instead of being better in mind she became worse; alarm arose both for her own life and that of her child; and as bodily recovery progressed from child-birth, the state of her mind seemed to become worse.

"She was removed to her seat in the country. Seven months had elapsed since her confinement, when I was requested to see her. I found her with such change of features, that in after-life I should scarcely have recognised her; in despair about her faults, her incapability of saying or doing what was right; considering herself a dirty and defiled creature, and showing the strongest disposition to suicide, towards which she had made many attempts. At times violence and destructiveness were mixed with the sad, deeply melancholy general expression. Her family, whom she dearly loved, were fiends in her imagination!

"After seeing the patient, and making careful inquiries, I said and felt satisfied this was one of the cases in which opiates had the best effect, and entered fully with the gentleman whom I met (a late most eminent general practitioner) into the reasons for such practice. I recommended 30 drops (considering the duration of the disease) of the solution, (two-thirds of a grain,) to be given every night at bed-time. I said, it will at first perhaps make her feel sick, or cause head-ache, but it will *cure her!* only remember, I do not expect any *marked good effect in less than a month.* Some days after, in consequence of a fresh attempt at self-destruction, I saw the gentleman again; I again earnestly impressed upon him the propriety of perseverance, from my experience. In about a fortnight, meeting him incidentally, he said, 'Oh! we were obliged to give up your medicine after a few days, it made her sick!' I made no answer, and considered my responsibility to be at an end.

"I lost sight of the case for the best of all reasons: soon after this conversation I was attacked myself by an illness so severe as to leave no expectation on my own mind, or hopes in my family, of my recovery, and of course all professional business was at an end. The first day in January (the last visit to the case had been early in October), on which I for the first time left my bed-room, a gentleman called on me to express the urgent wishes of the lady's family, that I should take the entire and sole charge of the patient. My health was so injured that I could then attend no one; but urged by importunity, I said I would write to the husband of the lady, if my health enabled me to return to my profession. At the end of January, I returned, feeble indeed, but able to commence my duties, and in the last week of that month the patient was placed under my charge. I found her, I thought, better in bodily health than on the former occasion, but everything in her room was boarded up which could be easily destroyed, and the female attendant informed me, that on that morning the patient had destroyed all the crockery by wilfully overturning the table, and that during the preceding nine weeks she had never slept without some restraint, as her tendency to self-destruction was well marked. It was my first desire to remove her home: it appeared to me that she would be as well with attendants in her own large house in London as in a much smaller one in the neighbourhood of the Regent's Park; but I was prevented by her house in London being dismantled, and a fortnight elapsed before this could be carried into effect. In the meantime I held out the return home as a reward for taking her medicine, which was the same in dose, and identical in composition, with that which I had ordered five months before. With the exception of occasional purgative medicine, no alteration was made during the continuance of the illness.

"In about a month after her return home, she was so much better that her children were restored to her. This was at the end of February. She then visited different relations, and on the 24th of May in that year was well enough to go to the Queen's drawing-room, all idea of serious illness being at an end.

"With a view of restoring her health, her kind friends, notwithstanding some difficulties from other engagements, resolved to try a tour on the continent. She remained well, but at times melancholy resumed its throne for a short time, and she returned to England not better than when she left home. Here I must be understood: she was in no state that approached the one in which I found her, but she was still low in spirits. This continued for about three months after her return, but at no time was there anything in her conduct and behaviour which could prevent her being a free agent. There were only shades of depression.

"From this time, her medicine being continued in half-doses, she entirely recovered, and when she died in child-bed (the third pregnancy since her illness), from an unusual accident in parturition, it was a consolation to all who knew her that she had retained for six years uninterruptedly her excellent sense and good spirits, even to a few minutes before her death."—Pp. 175—179.

Dr. Seymour does not entertain a high opinion of the advantages of travelling in these cases of melancholia. He conceives that it presents to an impaired mind a succession of objects too quickly, and, consequently, a change of place and variety are eminently injurious as a means of cure until the melancholy hallucinations are completely at an end.

On the important subject of occupation, Dr. Seymour has given us the advantage of his great experience. He maintains, that when amusement or occupation can be effected without compulsion, great benefit results. In this respect the pauper lunatics have the advantage of the more opulent and better class of insane patients. In large county asylums, it is not difficult to induce a great number of the inmates to engage in regular daily occupations—some in attending to the economy of the establishment, and others employed in the cultivation of the land attached to the asylum. This, superadded to the generous diet they enjoy, and regular habits they are obliged to conform to, often effect wonders in re-establishing mental health.

In the best regulated of our private establishments for the treatment of insanity, one of the most important elements of cure is in providing for the patients regular and steady occupation for the mind and body. It is, however, no easy thing to induce patients so situated to appreciate the advantages of occupation, or to induce them to engage in any regular employment.

In cases of mania, accompanied with a cessation of, or irregularity of the monthly function, Dr. Seymour thinks highly of the application of leeches to the cervix-uteri, immediately before the expected period of the discharge.

We make no apology for the quoting *in extenso* the author's description of a character of mental derangement which often developes itself at the critical age of 60, and which is considered dependent upon the circulation in the brain. He says—

"As life advances the mechanical portion of the fabric would be most likely to decay; and this we find to be the case. The arterial system loses its elasticity by the alteration of its coats; sometimes the destruction of its internal coat by softening, sometimes by the deposition of bone in the otherwise elastic tissue. The deposition of bone, often very considerable in the larger arteries, especially the aorta and radial, nearly obliterates the structure of the minuter arteries. This, in the smaller ramifications, gives rise to softening in the parenchyma of the brain, while in the larger arteries of the brain it occasions rupture of the vessels, whenever sudden and very considerably increased action of the heart is produced; but where disease does not go to this extent, it is clear great irregularity takes place as to the uniform pressure of blood in the vessel: hence, perhaps, the natural relief by the pouring out of fluid; or the brain itself, no longer equably and regularly nourished, becomes impaired in its perception in a similar way to that in which organic actions in other parts of the body are impaired by a deficiency of proper nutriment: whether this be or not the proper explanation, the facts are as follows:—

"The patient becomes morose and exceedingly distressed about trifles, and sometimes there is a melancholy foreboding of mischief. As time advances, the patient becomes oppressed with the feeling that it would be better and easier to escape from life itself, than to undergo the horrors that he experiences. Now, it is conspiracy of his dearest and best friends to deceive or destroy him; often, he considers he labours under some great moral imputation, and takes up the remembrances of youth for the purpose of establishing some terrible stain on the character which he is now, for the first time, fully aware of. Sometimes it is his affairs which are inextricably involved. It has occurred to me to see several of these cases of melancholy in advanced life, where the principal alarm was against personal arrest: although, at the same time, there was a large balance at the bankers', and an income equivalent to five times the expenditure, yet at any knock at the door the patient would expect a bailiff; or he passed hours in the deepest lamentations over bills and bonds of which he had been cheated, and which all the time reposed safely in his strong box. When these supposed missing securities were shown him, he would declare they were not the real ones, but forged for the purpose of deception. Intervals of ease, however, take place under these circumstances; they are frequent at the commencement, but gradually become less. Such cases very frequently terminate in suicide.

"The utmost quiet and tranquillity are necessary. The patient ought never to be left either by night or day, for suicidal propensities in these particular instances are extraordinarily ingenious. An instance occurred in a private asylum not many years ago, where a patient strangled herself when a nurse was sitting within four yards of her bed. The moral management, as it is termed, consists in a variety of amusements, which call for the least exercise of mental power; gentle riding on horseback; and, in doors, cards, billiards, backgammon, or any of those numerous engagements which occupy without employing the mind. The medical treatment which I have seen successful is entirely in conformity with the explanation which I have endeavoured to give. The patient, unless he be very plethoric, in which case he may require venesection, should be cupped at the back of the neck, to the amount of about six ounces the first time. This will relieve, not the plethoric condition of the vessels, but the unequal distribution of the blood; and every six weeks, as nearly as possible at the same period, the patient should lose about four ounces more. Some saline purgative medicine should be administered every morning, sufficient to procure two watery evacuations, and a quarter of a grain of morphia every night, increased after a time to half a grain, to relieve in this instance the effect, and not the cause, of a disordered organization. Neither warm nor cold baths appear to be of any use in this particular form of the disease.

"If, which is not unfrequently the case, the stomach be disordered, the tongue red and shining, symptoms of an irritated state of the mucous membrane of the intestinal canal, especially of the stomach, the patient may take twice a day such a dose of the medicinal prussic acid as may be considered by his adviser to be necessary, and from which often the greatest benefit is derived: and here particular attention is required to those periodical discharges which establish themselves as life advances. If the patient has been subject to hemorrhoids, which have ceased to discharge, leeches may be applied to the anus, instead of cupping to the back of the neck; if the urine be small in quantity, as is not unfrequently the case, the use of the preparations of nitre, with nitric æther and tincture of squill, may be taken twice in the day, in the place of the prussic acid recommended under a different condition of the system.

"In every case the diet must be moderate, and if animal food is taken it should be every alternate day, fish and farinaceous food being given on the intermediate days. On the subject of wine it is impossible for me to lay down any rule, because the same recommendation cannot apply to a man who through life has been very temperate, as to one who probably has habitually drank a bottle daily during the preceding forty years of his life. The smallest possible quantity must be allowed that can be reconciled with the previous habits of the patient. It has occurred to me to see more than one of these cases where the disease has been attributed to that parent of all evil, the liver, and the patient has been sent to drink the stimulating saline waters of Carlsbad, or even those much less stimulant of Wiesbaden; and even in more than one instance the factitious waters of Brighton. The use of these, thus *improperly* employed, has been followed by the most furious insanity; at other times paralytic seizures have ensued with fatal effect, and the worst part of these cases is, they have been invariably preceded by amelioration in the melancholy disposition of the patient, and frequently by a flow of spirits and eagerness of manner which, when contrasted with the former symptoms, have raised the most sanguine hopes of relations, which were speedily to be consigned to despair. The sedative treatment alone is perfectly useless; it is in a secondary view that it is useful, in diminishing the intensity of an effect, while the cause is relieved by the regulated and periodical treatment already related."—Pp. 186—191.

We can add nothing to the above graphic delineation. It is from the pen of a master-mind, and its truth—powerful truth! must be apparent to every experienced mind.

Dr. Seymour directs the attention of the profession to the frequent association of insanity with disease of the lungs. In these cases he has generally found the acetate of morphia produce little or no effect.

In cases of derangement of mind, connected with the refusal of food, the author suggests whether this melancholy and often most distressing form of madness may not arise from a contracted state of the stomach, secondarily to disease of the brain? We have no doubt that, in many of these cases, not only when the patient refuses food, but when he does so from a suspicion of its being poisoned or drugged, there exists some disease of the stomach itself. *Post mortem* examinations establish the truth of Dr. Seymour's suggestion; we have, under these circumstances, generally applied our remedies to the gastric organ itself, and generally have found, that in a ratio to our being enabled to restore it to a condition of health, has this symptom subsided. A blister applied to the region of the stomach has often entirely removed an obstinate disinclination for food.

Dr. Seymour points out another symptom, often associated with melancholy, as deserving of scientific inquiry. He refers to the extraordinary secretion from the salivary gland which takes place in many of these cases, and which he considers as very debilitating in its effects.

Dr. Seymour enters very fully into the consideration of the pathology of insanity, particularly in reference to the organic changes which are supposed to take place in the brain in cases where the mind is affected. He concludes his inquiry by observing, that—

"My conviction is, that hitherto no distinct relation has been decidedly established between mental derangement and organic disease, except the certainly fatal event when organic disease exists in conjunction with mental derangement. Thus a wide field is open for the observation of those who have the care of lunatics, in endeavouring to discover whether any or what lesion or change exist in the brain itself, as a cause for the long and hitherto inexplicable duration of this dreadful malady, especially in cases of hereditary insanity; this last being the most curious, as likely to lead to the most important results, especially as to the great point, whether this predisposition, as is the case with gout, scrofula, and lues, is transmitted through the blood, or through some hitherto ill-defined impression on the solids of the body, the configuration of the brain and its envelopes."—P. 208.

Dr. Seymour is supported in this view of the pathology of insanity by many of the brightest names in the annals of medicine. We are by no means disposed to materialism, although readily admitting the influence of matter on the manifestations of mind and spirit. We cannot shut our eyes to what we conceive to be obvious and important truths, yet we are willing to admit, that much injury to the mind itself may result from attributing too great a power to mere matter, and undervaluing the influence of pure spiritual operations.

On this interesting point, we do not hesitate in quoting a passage from the pen of a physician, whose views on this matter corresponded with those entertained by the author of the essay. In justice to Dr. Seymour, we refer to Dr. Cowan's remarks. This intelligent "spiritualist" observes —

"'While fully admitting that all mental and moral manifestations are effected through the medium of a special organization, and therefore dependent for their exercise on the health and integrity of the instrument, we have felt conscious that the agency of mind upon matter was virtually, though not always nominally, disregarded, and that the actual increase of our knowledge, resulting from cranioscopical examination, is really much less than what is confidently assumed. We do not attribute the increasing prevalence of juster impressions so much to the progress of science, as to clearer apprehensions of revealed truth. It is the stern standard of moral responsibility which christianity inculcates, enlightening our internal experience, which makes man acquainted with his compound and mysterious nature, and prevents his descent to the level of a shallow materialism. No man can doubt, if deducing his conclusions from the knowledge of his own heart, that his mental aberrations from what is right are *moral*, not *cerebral* delinquencies; and if so, why should we shrink from admitting the possibility of morbid manifestations, sometimes originating in a perverted spiritual principle, as well as in diseased organization?'"

On the incurability of insanity, when complicated with true epilepsy, Dr. Seymour's experience accords with that of Esquirol, who says,— "*L'épilepsie compliquée d'aliénation mentale ne guérit jamais.*" With the recurrence of epilepsy, the mind is often partially restored to health, giving to the relatives faint hopes of recovery. Alas! these hopes are but of transient duration—the clearing up of the intellect is but a harbinger of speedy dissolution ! In cases in which morphia is not admissible, Dr. Seymour trusts to tartar emetic, shower bath, counter-irritants, and purgatives. In cases of insanity supervening upon concussion of the brain, blood-letting, particularly cupping in the neighbourhood of the head, setons in the neck, and calomel, succeeded by purgatives, is the plan of treatment recommended by the author.

Our analysis of Dr. Seymour's valuable "thoughts" on the subject of mental derangement, has already exceeded our limits, and we must lay down the critical pen. In doing so, we would observe, that the last report of the Commissioners in Lunacy affords ample testimony in favour of Dr. Seymour's sedative plan of treatment. We had marked many passages in the report for quotation, but, at present, we can do no more than refer the reader to the opinions given by men of great practical experience in the treatment of insanity, in reply to questions submitted to them by the commissioners. It will be found that the preponderance is greatly in support of the view which Dr. Seymour takes of the medical treatment of affections of the mind.

